# The foremost and greatest barrier to end-stage heart failure treatment: the impact of caregiver shortage

**DOI:** 10.1007/s10047-024-01463-x

**Published:** 2024-08-07

**Authors:** Shunsuke Saito, Daisuke Yoshioka, Takuji Kawamura, Ai Kawamura, Yusuke Misumi, Yasuhiro Akazawa, Fusako Sera, Kaori Kubota, Takashi Yamauchi, Yasushi Sakata, Shigeru Miyagawa

**Affiliations:** 1 Department of Cardiovascular Surgery, Osaka University Graduate School of Medicine, 2-2 Yamada-Oka, Suita, Osaka 565-0871 Japan; 2 Department of Cardiovascular Medicine, Osaka University Graduate School of Medicine, Suita, Osaka 565-0871 Japan; 3 Department of Nursing, Osaka University Hospital, Suita, Osaka 565-0871 Japan

**Keywords:** Left ventricular assist device, Home therapy, Caregiver, Heart transplantation

## Abstract

We examined the number of patients abandoning cardiac replacement therapy due to the inability to secure a designated caregiver. At Osaka University Hospital Heart Center, when we receive a consultation for a patient with severe heart failure from another hospital, a heart failure team makes a visit to the referring hospital as soon as possible. We retrospectively analyzed this hospital-visit database. We received 199 severe heart failure consultations from 2016–2023. Issues identified during hospital visits included age ≥ 65 years (8%), inability to confirm the patient’s intention (8.5%), and explicit refusal of therapy (2.5%). Medical problems included multiple organ failure (18.1%), obesity (13.1%), diabetes (9.5%), malignancy (5.5%), chronic dialysis (1.0%), and other systemic diseases (12.6%). Adherence problems included poor medication compliance (3.5%), history of heavy drinking (2.5%), and smoking (2.0%). Social problems included inadequate family support in 16.1% of patients. Of the 199 patients, 95 (48.0%) proceeded to a heart transplant and LVAD indication review meeting at Osaka University Hospital. The remaining 104 patients (52.0%) did not proceed to the meeting. Reasons included improvement of heart failure with conservative treatment in 37 cases (35.6%), death before discussion in 21 cases (20.2%), medical contraindications in 18 cases (18.3%), lack of caregivers in 18 cases (18.3%; 9.5% of 199 cases), and patient refusal in 5 cases (4.8%). Approximately 10% of patients consulted at Osaka University Hospital Heart Center for severe heart failure abandoned cardiac replacement therapy due to the lack of caregivers.

## Introduction

Heart transplantation is widely regarded as the optimal treatment for advanced heart failure [1]. However, in Japan, patients with severe heart failure face an average wait time of approximately five years for a heart transplant due to a severe shortage of donors [2]. Consequently, the majority of patients on the waiting list rely on a left ventricular assist device (LVAD) for support, often for extended periods, as a bridge to transplantation (BTT). The HeartMate 3 LVAD was approved for use as destination therapy (DT) in Japan and covered by insurance starting April 30, 2021, allowing LVAD treatment without the necessity of subsequent heart transplantation [3].

The critical role of caregiver support in successful long-term LVAD assistance is well recognized [4–6]. The Japanese Circulation Society’s criteria for heart transplantation stipulate that “at least one (preferably two) adult immediate family member or spouse must provide support” [7]. In addition, there exists an “unwritten” requirement for home care of patients with implantable LVADs, mandating caregiver presence 24 h a day, 365 days a year. The criteria for LVAD as DT have been relaxed to not require cohabitation with a caregiver after six months [8], and the criteria for BTT have been similarly relaxed to match DT requirements from April 2024 [9]. It was further specified that 24 h service is required from hospitals, not caregivers. Despite these relaxed conditions, the requirements for heart transplantation have not been modified. This implies that, in Japan, patients cannot proceed to heart transplantation or LVAD treatment unless they have a caregiver who can live with them for at least 6 months.

This stringent requirement creates a situation where patients with severe heart failure, who are medically in need of heart transplantation or LVAD, are unable to receive adequate treatment due to the inability to secure a dedicated caregiver. However, there has been no previous research quantifying the number of patients who forgo cardiac replacement therapy due to this issue. In this study, we investigated the number of patients who abandon cardiac replacement therapy because they cannot secure a designated caregiver.

## Patients and methods

Ethical committee approval was obtained for this retrospective study. At the Osaka University Hospital Heart Center, when a consultation is received for a patient with severe heart failure from another hospital, a heart failure team visits the referring hospital as soon as possible. The visiting team typically consists of a heart failure physician, a cardiac surgeon, and a transplant coordinator. At the referring hospital, after discussing the patient’s condition with the attending physician, the patient undergoes an examination, and future plans are deliberated with the attending physician, the patient, and the family. Following these discussions, the patient’s treatment plan is determined: immediate transfer to Osaka University Hospital or continuation of treatment at the referring hospital, continuation of medical management, or immediate initiation of mechanical circulatory support. The patient’s treatment goal is also discussed: heart transplantation, implantable LVAD as a destination therapy, or medical treatment/temporary mechanical support as a bridge to recovery.

Patient data are prospectively gathered in the heart failure consultation database. Parameters recorded in the database include the date of referral, date of hospital visit, name and location of the referral hospital, patient’s age and gender, diagnosis, interagency registry for mechanically assisted circulatory support (INTERMACS) profile, circulatory support at the time of the visit, and the patient’s problems that the visiting team considered potential obstacles to proceeding to heart transplantation or implantable LVAD treatment. The decision reached by the patient, family, attending doctor, and the visiting team is also documented.

In the present study, we retrospectively analyzed the data of patients who were consulted at the Osaka University Hospital Heart Center for heart failure treatment. Data were extracted from the heart failure consultation database and patient charts recorded in the hospital’s electronic medical records.

## Results

Between January 2016 and December 2023, the Osaka University Hospital Heart Center received 199 consultations for severe heart failure patients from other hospitals. Table 1 summarizes the demographics of these patients. The mean age was 49.7 ± 12.2 years, with 28.6% being female. The locations of the referral hospitals were within Osaka prefecture for 34.7% of the patients, within the Kinki region outside Osaka for 19.6%, and outside the Kinki region for 46.7%. The leading causes of heart failure were idiopathic cardiomyopathy (51.8%), followed by ischemic cardiomyopathy (15.1%), myocarditis (12.6%), secondary cardiomyopathy (11.1%), and acute myocardial infarction (7.5%). Many of the patients were classified as INTERMACS profile 3 (30.2%) or 4 (26.6%), with 24.6% and 18.6% in profiles 1 and 2, respectively. At the time of the visit, 35.2% of the patients were supported by continuous intravenous inotropes, 11.1% by intra-aortic balloon pumping, 18.1% by extracorporeal membrane oxygenation, and 11.0% by Impella or temporary LVAD. All the patients in INTERMACS profile 4 were still hospitalized at the referring hospital and could not visit our outpatient clinic at the time of consultation.

**Table 1:** Patients background

Mean age, years (range)	49.7 ± 12.2 (16 – 72)
Female gender, no (%)	57 (28.6)
Referral hospital location, no (%)	
Osaka prefecture	69 (34.7)
Kinki region other than Osaka	39 (19.6)
Outside Kinki region	91 (46.7)
Diagnosis, no (%)	
Idiopathic cardiomyopathy	103 (51.8)
Ischemic cardiomyopathy	30 (15.1)
Myocarditis / post-myocarditis cardiomyopathy	25 (12.6)
Secondary cardiomyopathy	22 (11.1)
Acute myocardial infarction	15 ( 7.5)
Unknown	4 ( 2.0)
INTERMACS profile, no (%)	
1	49 (24.6)
2	37 (18.6)
3	60 (30.2)
4 or higher	53 (26.6)
Circulatory support, no (%)	
None	49 (24.6)
Inotropes	70 (35.2)
IABP	22 (11.1)
ECMO ± IABP	36 (18.1)
IMPELLA	12 ( 6.0)
IMPELLA + ECMO	4 ( 2.0)
Temporary LVAD	6 ( 3.0)

*ECMO* extracorporeal membrane oxygenation, *IABP* intra-aortic balloon pumping, *INTERMACS* interagency registry for mechanically assisted circulatory support, *LVAD* left ventricular assist device

Table 2 summarizes the obstacles identified by the visiting team that could impede the patient’s listing on the heart transplant waiting list or proceeding to destination therapy with an implantable LVAD. Eight percent of the patients were 65 years or older, which is an exclusion criterion for heart transplantation in Japan. The willingness of 8.5% of the patients to accept heart transplantation or LVAD treatment could not be ascertained, and 2.5% of the patients explicitly refused heart transplantation or LVAD after a detailed explanation from the visiting team about the advantages and disadvantages of undergoing heart transplant and LVAD treatment. Medical problems included multiple organ failure (18.1%), obesity (13.1%), poorly controlled diabetes (9.5%), a history of or current malignancy (5.5%), chronic dialysis (1.0%), and other systemic diseases (12.6%). Issues related to adherence were also noted, such as poor medical compliance (3.5%), a history of heavy alcohol consumption (2.5%), and current smoking (2.0%). Social problems included inadequate familial support in 16.1% of the patients, and one patient had a criminal record (history of illegal drug abuse).

**Table 2:** Patients’ problems at the time of referral

Age ≥ 65y, no (%)	16 ( 8.0)
Unable to ascertain the patient’s will, no (%)	17 (8.5)
Clear refusal of heart transplantation, no (%)	5 ( 2.5)
Medical problems, no (%)	
Multiple organ failure	36 (18.1)
Obesity	26 (13.1)
Poorly controlled diabetes	19 ( 9.5)
Current or history of malignancy	11 ( 5.5)
Chronic dialysis	2 ( 1.0)
Other systemic diseases	25 (12.6)
Problems of the patient’s adherence to heart failure treatment, no (%)	
Poor compliance	7 ( 3.5)
Heavy alcohol-drinking history	5 ( 2.5)
Current smoking	4 ( 2.0)
Social problems, no (%)	
Inadequate familial support	32 (16.1)
Criminal record	1 ( 0.1)

**Fig. 1:**
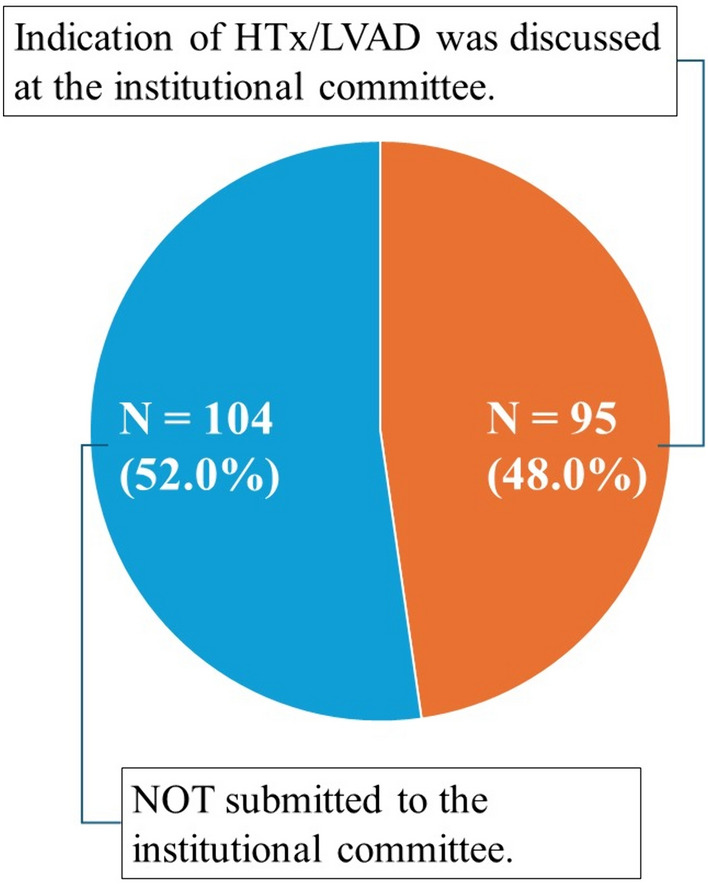
Percentage of cases that proceeded to the Osaka University heart transplantation/LVAD Indication Review Committee and those that did not

**Fig. 2:**
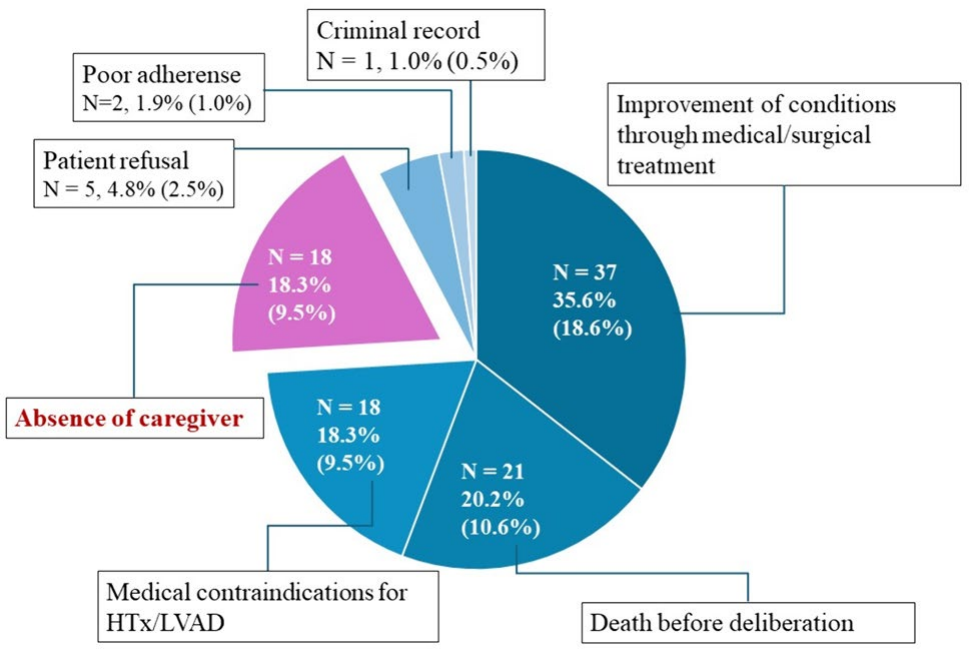
Reasons for not proceeding to the heart transplant/LVAD Indication Review Committee

After the hospital visit by the heart -failure team, 95 of the 199 patients (48.0%) proceeded to discussions at Osaka University Hospital’s institutional review committee for heart transplantation/LVAD indication. None of these cases had their heart transplant/LVAD applications rejected due to lack of caregiver. The remaining 104 patients (52.0%) were not submitted to the institutional committee (Fig. 1). Reasons for not proceeding to the committee are summarized in Fig. 2. There was potential for further medical or surgical heart failure treatment in 37 patients (35.6%), and their conditions improved without heart transplantation or LVAD. Twenty-one patients (20.2%) were too ill and died before deliberation. Eighteen patients (18.3%) had clear medical contraindications for heart transplantation or LVAD. Eighteen patients (18.3%) could not secure a caregiver, representing 9.5% of the entire cohort. Five patients (4.8%) refused heart transplantation or LVAD. Two patients could not proceed due to poor adherence, and one due to a criminal record.

## Discussion

In the present study, it was revealed that almost 10% of the patients consulted at the Osaka University Hospital Heart Center for advanced heart failure treatment had to abandon heart transplantation or LVAD due to the lack of caregivers. The primary reason for not advancing to discussions at the institutional review committee for heart transplantation or LVAD indication was the improvement of conditions through medical or surgical treatment. The second leading cause was patient death before deliberation. For many patients who died before the deliberation, the consultation appeared to have been too late. Earlier consultations might have led to higher survival rates. The absence of caregivers was the third most common reason for not proceeding to discussions at the institutional review committee. This is the first study to examine the availability of caregivers for patients with severe heart failure in Japan.

Although the importance of caregiver support for end-stage heart failure treatment is well known [4–6], previous studies focusing on caregivers of patients who underwent heart transplantation or LVAD implantation in Japan are rare [10, 11]. Suzuki et al. studied changes in the quality of life (QOL) in 32 LVAD patients and 24 caregivers before and after LVAD implantation. In LVAD patients, the QOL improved significantly post-implantation if there were no significant LVAD-related complications. However, for caregivers, while their anxiety reduced, their QOL remained unchanged before and after LVAD. Their study suggested that social QOL and activity levels did not improve post-implantation, indicating that the social participation and activities of LVAD patients and their caregivers are limited. They discussed that the requirement for caregivers to always accompany the patient prevents both LVAD patients and caregivers from fulfilling their normal social roles [10].

Kato et al. also examined the impact of LVAD implantation on caregivers’ QOL in a longitudinally quantitative study. They showed that the mental QOL of caregivers improved after LVAD implantation, but it remained lower than that of the general Japanese population. The caregiving burden increased post-discharge compared to pre-implantation, and this burden was an independent predictor of lower mental QOL for caregivers. They reported that caregivers felt their relationships with friends and other family members suffered due to the caregiving responsibilities. In addition, the study revealed that the lower QOL of caregivers was associated with their unemployed status [11]. It is often the case that caregivers must quit their jobs because of the rule that they must accompany LVAD patients at all times. Kato et al.’s study shows that the QOL of caregivers who quit their jobs for caregiving is significantly impaired. Conversely, many situations arise where both the patient and the caregiver cannot financially sustain themselves if the caregiver quits their job, forcing patients to abandon the LVAD option. These patients are included in the 10% who abandoned LVAD treatment due to the lack of caregivers in the present study.

### Implications for clinical practice and future research

**Fig. 3:**
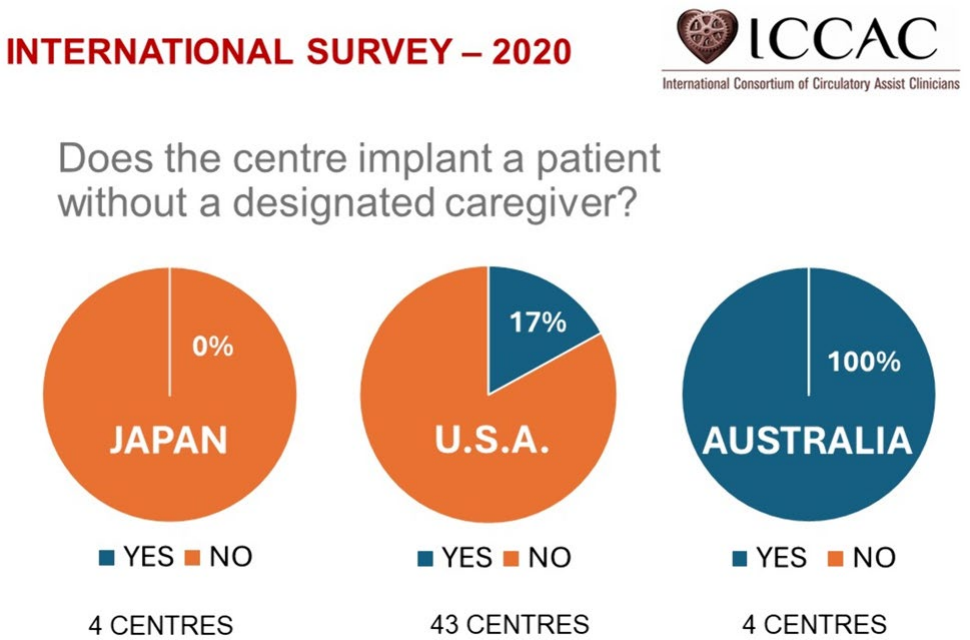
Results of an international survey conducted by the International Consortium of Circulatory Assist Clinicians (ICCAC) in December 2020, which asked, “Does the center implant LVAD in a patient without a designated caregiver?” (permission to publish obtained from ICCAC)

The present study revealed that a significant percentage of severe heart failure patients are forced to abandon LVAD or heart transplant treatment due to the requirement that caregivers must attend to LVAD patients 24 h a day, 365 days a year. This situation undermines patients’ rights to equal access to the best possible care. Figure 3 presents the results of an international survey conducted by the International Consortium of Circulatory Assist Clinicians (ICCAC) in December 2020, which asked, “Does the center implant LVAD in a patient without a designated caregiver?” (permission to publish obtained from ICCAC). In the United States, 17% of LVAD centers replied that they would implant LVAD in a patient without a caregiver, and in Australia, 100% (4/4) of centers replied that they would do so. Japan is among the minority of countries that completely prohibit the placement of implantable LVADs in patients without caregivers [12] and is the only country that requires the caregiver to be physically present with the LVAD patient at all times.

Given that the HeartMate 3 has become the mainstream implantable LVAD and has significantly reduced events such as sudden pump stoppages and severe strokes [13, 14], it is imperative to reconsider the necessity of this stringent caregiver requirement. To repeal this rule, it is essential to understand how strictly current LVAD patients and their caregivers adhere to this requirement and how much their quality of life (QOL) is impaired by it. Furthermore, it is necessary to provide clear data on the incidence of serious LVAD-related accidents and the extent to which the presence of a caregiver prevents these incidents. Future research should focus on several key areas:

1. Adherence to caregiver requirements: studies should investigate the actual adherence levels of current LVAD patients and their caregivers to the 24/7 rule and assess the impact on their QOL.

2. Quality of life assessment: further research should evaluate the QOL of both patients and caregivers under the current caregiving requirements, using robust and validated tools.

3. Safety and incident data: collect and analyze data on serious LVAD-related incidents to determine how often caregiver intervention prevents accidents and whether the stringent caregiver presence requirement is justified.

4. Policy and practice comparison: comparative studies with countries that have more relaxed caregiver requirements can provide insights into the impact of different policies on patient outcomes and safety.

By addressing these areas, we can develop evidence-based guidelines that balance patient safety with quality of life, potentially leading to policy changes that improve access to LVAD and heart transplant treatments for patients in Japan.

### Study limitations

There are several limitations to this study, including its retrospective nature and the relatively small number of patients included. The patients included in this study were limited to those who were referred from other hospitals to Osaka University, and the hospital visits were conducted by the heart failure team for the treatment of severe heart failure. These patients do not necessarily reflect the overall situation of patients with severe heart failure in Japan. The detailed reasons for the inability to secure caregivers were unavailable because they were not documented in the heart failure consultation database, although most of reasons were the physical absence of a caregiver and economic factors. The outcomes of patients who were not transferred to Osaka University Hospital were unavailable. It is highly likely that the prognosis of patients who required a heart transplant or LVAD but were forced to abandon these options due to the lack of a caregiver would have been significantly poor. Caregiver requirements for home LVAD treatment were somewhat relaxed from April 2024. This study was conducted before that rule change, and the situation may change slightly in future. These limitations underscores the need for a more comprehensive, multicenter prospective study to capture a broader range of patient outcomes and provide a more definitive assessment of the impact of caregiver requirements on access to advanced heart failure treatments.

## Conclusion

In the present study, it was revealed that approximately 10% of patients consulted at Osaka University Hospital Heart Center for severe heart failure abandoned cardiac replacement therapy due to the lack of caregivers. This finding highlights a critical barrier to accessing life-saving treatments such as heart transplantation and LVAD.

Further studies are needed to determine if the current rule requiring a designated caregiver to be constantly present with the LVAD patient is truly necessary. Such research should explore alternative support structures and the potential for technological solutions to reduce the burden on caregivers. By reassessing and potentially modifying these requirements, we can work toward ensuring that all patients have equitable access to the best possible care for severe heart failure.
